# Cross-Sectional and Longitudinal Associations Between Weight Perception and Cardiometabolic Health Among Adolescents Followed Into Emerging Adulthood

**DOI:** 10.5888/pcd23.250386

**Published:** 2026-06-25

**Authors:** Mikelina Djekovic, Abdulghafoor Alani, C. Blair Burnette, Rachael K. Nelson, Julie C. Lumeng, Samantha L. Hahn

**Affiliations:** 1 Central Michigan University, College of Medicine, Mount Pleasant, Michigan; 2 Department of Psychology, Michigan State University College of Social Science, East Lansing, Michigan; 3 School of Health Sciences, Central Michigan University School of Health Sciences, Mount Pleasant, Michigan; 4 Department of Developmental and Behavioral Pediatrics, University of Michigan Medical School, and Department of Nutritional Sciences, University of Michigan School of Public Health, Ann Arbor, Michigan

## Abstract

**Introduction:**

Several US public health and clinical initiatives notify adolescents of body mass index (BMI) or weight status; however, few studies have examined whether weight perception is independently associated with objective cardiometabolic markers. We assessed cross-sectional and longitudinal associations between weight perception and cardiometabolic health among adolescents followed into emerging adulthood.

**Methods:**

We analyzed wave 4 (mean [SE] age, 19.2 [0.04] y) and wave 7 data collected 3 years later from the NEXT PLUS Generation Health Study, a nationally representative cohort of US adolescents followed into emerging adulthood (analytic n = 454 at wave 4; n = 330 at wave 7). To assess cross-sectional and longitudinal associations between weight perception (perceived their weight as overweight vs did not perceive their weight as overweight) and cardiometabolic outcomes (fasting blood glucose, hemoglobin A_1c_, high-sensitivity C-reactive protein, triglycerides, total cholesterol, high-density lipoprotein cholesterol, low-density lipoprotein [LDL] cholesterol, systolic blood pressure, diastolic blood pressure, and waist circumference), linear regressions adjusting for sociodemographic variables were performed with and without additional adjustment for BMI.

**Results:**

In cross-sectional models adjusted for sociodemographic characteristics and BMI, participants who perceived their weight as overweight had higher adjusted mean total cholesterol (mean difference, 12.3 mg/dL; *P* = .045) and LDL cholesterol (mean difference, 8.3 mg/dL; *P* = .04). In longitudinal models, perceiving one’s weight as overweight was not associated with cardiometabolic outcomes after adjustment for BMI.

**Conclusion:**

Perceiving one’s weight as overweight is associated with worse cardiometabolic health. Health professionals should consider the possible implications of policies aimed at informing adolescents of their BMI status before implementation. Future studies are warranted to explore the causal pathway of this association.

**Summary**

**What is already known on this topic?**

Weight perception is associated with changes in health behaviors and psychological outcomes, but evidence linking weight perception to objective cardiometabolic markers is limited.

**What is added by this report?**

In adolescents followed into emerging adulthood, perceiving one’s weight as overweight was cross-sectionally associated with higher total and low-density lipoprotein cholesterol after body mass index (BMI) adjustment, but no longitudinal associations remained after BMI adjustment.

**What are the implications for public health practice?**

Practices aiming to notify people of their weight status do not have the intended benefits of improving health and may in fact worsen cardiometabolic health.

## Introduction

Weight perception refers to how people perceive their body size, regardless of measured weight status. Public health and clinical practices often include notification of body mass index (BMI) or weight-status categories. For example, school-based BMI screening and parent-notification programs have been implemented in several states, and it is routine for pediatric patients to be weighed and told their BMI status at well-child visits (1–3). These approaches are often intended to promote health behavior change and reduce cardiometabolic risk, although evidence that BMI notification alone achieves these goals is limited (4,5).

However, emerging evidence suggests that BMI-notification practices may not achieve intended outcomes and may have unintended adverse effects. Studies of school-based BMI screening and reporting have failed to reduce BMI and have increased body dissatisfaction, parental pressure, and peer stigmatization (6,7). Moreover, perceiving oneself as overweight, independent of actual weight, has been linked to greater disordered eating, lower physical activity, and fewer health-promoting dietary behaviors (8,9). Adverse effects are thought to arise from weight stigma, whereby people internalize negative views of their bodies, avoid stigmatizing environments (eg, gyms), or engage in unhealthy compensatory behaviors (4,8–14). Beyond behavior, weight stigma itself increases physiological stress and higher allostatic load over time (15). Thus, in a weight-stigmatizing society, perceiving one’s weight as overweight may harm health through both behavioral and biological pathways (15–19).

Research linking weight perception to objective cardiometabolic markers is limited, especially among adolescents transitioning to emerging adulthood. A prior study of adolescents with higher body weights and a study of Korean adults suggest that weight perception may be associated with elevated blood pressure during this transition (13,20). However, longitudinal biomarker data during the transition from adolescence to emerging adulthood remain scarce. Understanding whether weight perception is independently associated with cardiometabolic health is important for evaluating the assumptions underlying public health and clinical practices that may influence weight perception (21,22).

We investigated cross-sectional and longitudinal associations between weight perception and physiological markers of cardiometabolic health in a nationally representative cohort of adolescents followed into emerging adulthood. We hypothesized that participants who perceived themselves as overweight would have less favorable cardiometabolic outcomes than participants who did not, both at baseline and 3 years later, after adjustment for sociodemographic covariates and BMI (13,20).

## Methods

### Study sample

Data came from the NEXT PLUS Generation Health Study conducted by the Eunice Kennedy Shriver National Institute of Child Health and Human Development. NEXT was an annual survey administered to a nationally representative sample of US adolescents followed into emerging adulthood from 2010 through 2017. The original full NEXT Generation health sample included 10th graders selected from public, private, and parochial schools in all 50 states (N = 2,874). A subset of this sample (the PLUS sample, n = 560) also had anthropometric and cardiometabolic biomarkers collected at wave 4 (mean age, 19.2 y) and wave 7 (3 years later). Although cardiometabolic markers were also collected at wave 1, we analyzed waves 4 and 7 because our research question focused on the transition from adolescence to emerging adulthood. Further, given that our research question was reliant on weight perception and collection of cardiometabolic markers, our analytic sample was restricted to those who had data on weight perception at wave 4 and at least 1 cardiometabolic outcome at wave 4 or wave 7, resulting in analytic samples of 454 for cross-sectional analyses and 330 for longitudinal analyses; exact sample sizes for each analyses could vary because participants were included if they had at least 1 cardiometabolic outcome. Consent was obtained from parents/guardians and assent from the participating adolescents at the beginning of the NEXT Generation Health Study. Once participants turned 18, adolescent participants provided written informed consent for themselves. The NEXT PLUS Generation Health Study was reviewed and approved by the Eunice Kennedy Shriver National Institute of Child Health and Human Development Institutional Review Board.

### Measures

#### Weight perception

Participants were asked, “At this time, do you feel you are . . .” with the following response options: “very underweight,” “somewhat underweight,” “about the right weight,” “somewhat overweight,” “and very overweight.” We classified participants as perceiving their weight as overweight if they answered “somewhat overweight” or “very overweight”; all other responses (“very underweight,” “somewhat underweight,” or “about the right weight”) were classified as not perceiving their weight as overweight, consistent with prior weight-perception research (10,12).

#### Cardiometabolic health markers

We assessed 10 cardiometabolic measures: fasting blood glucose, high-sensitivity C-reactive protein (hs-CRP), triglycerides, total cholesterol, high-density lipoprotein (HDL) cholesterol, low-density lipoprotein (LDL) cholesterol, hemoglobin A_1c_ (HbA_1c_), systolic blood pressure, diastolic blood pressure, and waist circumference. All measures were assessed at wave 4, and all measures except blood pressure and waist circumference were assessed at wave 7. Blood samples were collected after a fast of at least 10 hours (ie, no food or drink except water and medication) to assess fasting blood glucose, hs-CRP, total cholesterol, HDL cholesterol, LDL cholesterol, and HbA_1c_. Blood pressure and waist circumference were measured by a trained researcher. Waist circumference was measured twice to ensure both measurements were within 1 cm of each other; if a discrepancy occurred, the measurement was repeated until 2 recorded values were within 1 cm of each other. All cardiometabolic outcomes were analyzed as continuous variables.

#### Covariates

The sociodemographic characteristics of the participants were collected in wave 1 of the survey and were treated as covariates in analyses, as was wave 4–measured BMI. Wave 1 covariates included self-reported sex (male or female), race and ethnicity (categorized by the survey response options as non-Hispanic Black, Hispanic/Latinix of any race, non-Hispanic White, and other race or ethnicity), socioeconomic status (SES), and age. SES was determined by using the Family Affluence Scale (FAS) as has been used in the past in this sample (23). This internationally used and validated scale categorizes participants into low, middle, or high SES based on number of cars the family owns, number of computers in the home, number of vacations in the past 12 months, and whether the adolescent has their own bedroom. A composite FAS score was calculated from responses to these 4 questions. Scores of 0 to 2 were categorized as low SES, scores of 3 to 5 as middle SES, and scores of 6 to 9 as high SES (24). BMI was calculated by using each participant’s measured height and weight and treated as a continuous variable. Height was measured with participants standing erect, with head position in the Frankfort plane; measurements were recorded at eye level. If hairstyle or a headscarf prevented the measuring piece from lying flush on the participant’s scalp, “interference height” was measured and recorded. Weight was measured via Healthometer Scale 498K; participants were asked to remove heavy layers of clothing or jewelry, and measurements were taken twice or until 2 readings were within 0.2 kg of each other. Participants weighing more than 500 lb were weighed using 2 scales. Sociodemographic covariates were chosen a priori based on existing literature showing associations between these variables and weight perception and cardiometabolic health outcomes (16,25–27).

### Statistical analysis

All analyses used inverse probability weighting to account for sampling bias and loss to follow-up and to make results generalizable to the original nationally representative sample (28). Univariate statistics were calculated for all model variables including weight perception, cardiometabolic outcomes, and covariates. Weighted independent-samples *t* tests assessed differences in baseline (wave 4) cardiometabolic outcomes between participants who did and did not perceive their weight as overweight at baseline. Linear regression models assessed associations between baseline (wave 4) weight perception and cardiometabolic outcomes at baseline (wave 4) and follow-up (wave 7). All regressions adjusted for sex, race and ethnicity, SES, and age. Longitudinal associations additionally adjusted for baseline levels of cardiometabolic outcomes. We ran analyses with and without adjustment for BMI for all regression analyses. All analyses were conducted using SAS version 9.4 (SAS Institute Inc). Results are presented as adjusted marginal means to increase interpretability and promote ability to compare with clinically significant values. Statistical significance was set at 2-sided *P* < .05. *P* values were not adjusted for multiple comparisons.

## Results

### Sociodemographic characteristics

Just over half of the sample was female (55.8%) (Table 1). Most of the sample was non-Hispanic White (62.0%), followed by Hispanic (24.8%), non-Hispanic Black (11.5%), and other race and ethnicity (1.8%). Approximately two-thirds of the sample were considered high SES (66.6%), with 30.3% middle SES, and only 3.2% considered low SES. Sociodemographic characteristics were generally similar by weight-perception group, except for sex and BMI. The mean (SE) BMI for participants who perceived their weight as overweight was 30.5 (0.69), compared with 23.0 (0.36) among participants who did not perceive their weight as overweight. More female than male participants perceived their weight as overweight (63.3% vs 36.7%).

**Table 1:** Demographic Characteristics of the Overall Sample and by Weight Perception, NEXT PLUS Generation Health Study, Wave 4, US^a^

Characteristic	Total population (n = 454)	Did not perceive overweight (n = 224)	Perceived overweight (n = 230)
**Total, %**	100.0	49.8	50.2
**Sex, % (raw n)**			
Female	55.8 (263)	48.2 (110)	63.3 (153)
Male	44.2 (191)	51.8 (114)	36.7 (77)
**Race and ethnicity, % (raw n)**			
Non-Hispanic Black	11.5 (83)	12.4 (44)	10.5 (39)
Hispanic/Latinx	24.8 (155)	21.6 (69)	28.0 (86)
Other race or ethnicity	1.8 (15)	3.1 (12)	0.5 (3)
Non-Hispanic White	62.0 (201)	62.9 (99)	61.0 (102)
**Socioeconomic status, % (raw n)^b^**			
Low	3.2 (31)	1.7 (7)	4.7 (24)
Middle	30.3 (160)	28.8 (81)	31.7 (79)
High	66.6 (259)	69.5 (134)	63.7 (125)
**Age, mean (SE), y^c^**	19.2 (0.04)	19.1 (0.08)	19.2 (0.04)
**Body mass index**	26.8 (0.49)	23.0 (0.36)	30.5 (0.69)

a Percentages and means were weighted by using inverse probability weights; raw n values are unweighted.

b Socioeconomic status (SES) was categorized by using Family Affluence Scale scores: low, 0–2; middle, 3–5; high, 6–9.

c SES denominators differ because of missing data. Age and body mass index were calculated at wave 4.

### Cross-sectional associations between weight perception and cardiometabolic health outcomes

#### Unadjusted *t* tests

Participants who perceived their weight as overweight had a higher mean hs-CRP than participants who did not perceive their weight as overweight (2.30 mg/L vs 1.16 mg/L; *P* < .001) (Table 2). Those who perceived their weight as overweight also had significantly higher mean triglyceride (102.5 mg/dL vs 79.9 mg/dL; *P* = .04), cholesterol (165.3 mg/dL vs 148.9 mg/dL; *P* = .007), and LDL cholesterol measurements (92.4 mg/dL vs 79.5 mg/dL; *P* < .001) and lower mean HDL cholesterol level (47.4 mg/dL vs 52.1 mg/dL; *P* = .03) compared with those who did not perceive their weight as overweight. Mean diastolic blood pressure for those who perceived their weight as overweight was 68.2 mm Hg, which was significantly higher than that of those who did not perceive their weight as overweight (64.7 mm Hg; *P* = .002). Similarly, mean waist circumference was significantly higher in those who perceived their weight as overweight than those who did not (96.6 cm vs 78.9 cm; *P* < .001). Mean HbA_1c_, fasting blood glucose, and systolic blood pressure did not differ statistically by weight perception.

**Table 2:** Weighted Unadjusted Means of Cardiometabolic Biomarkers, by Weight Perception, NEXT PLUS Generation Health Study, Wave 4, US^a^

Biomarker	Overall	Did not perceive overweight	Perceived overweight	*P* value^b^
HbA_1c_, %	5.35 (0.03)	5.35 (0.03)	5.35 (0.04)	.99
Fasting blood glucose, mg/dL	93.8 (0.7)	93.6 (1.1)	93.9 (0.9)	.84
High-sensitivity C-reactive protein, mg/L	1.76 (0.17)	1.16 (0.21)	2.30 (0.26)	<.001
Triglycerides, mg/dL	91.6 (5.8)	79.9 (6.3)	102.5 (9.0)	.04
Cholesterol, mg/dL	157.4 (3.3)	148.9 (3.3)	165.3 (5.1)	.007
High-density lipoprotein cholesterol, mg/dL	49.7 (1.1)	52.1 (1.2)	47.4 (1.8)	.03
Low-density lipoprotein cholesterol, mg/dL	86.2 (1.9)	79.5 (2.4)	92.4 (2.4)	<.001
Systolic blood pressure, mm Hg	112.6 (0.8)	113.1 (1.3)	112.0 (0.9)	.50
Diastolic blood pressure, mm Hg	66.4 (0.6)	64.7 (0.8)	68.2 (0.9)	.002
Waist circumference, cm	87.5 (1.2)	78.9 (1.0)	96.6 (1.7)	<.001

a Values are mean (SE) unless otherwise indicated. All calculations used inverse probability weighting for wave 4.

b *P* values are from weighted independent-samples *t* tests comparing weight-perception groups.

#### Cross-sectional adjusted linear regressions

When controlling for sex, race and ethnicity, SES, and age, those who perceived their weight as overweight had higher adjusted mean hs-CRP (2.10 mg/L vs 0.96 mg/L; *P* < .001), triglycerides (92.6 mg/dL vs 64.8 mg/dL; *P* = .01), cholesterol (164.0 mg/dL vs 145.5 mg/dL; *P* = .005), and LDL cholesterol (93.4 mg/dL vs 80.0 mg/dL; *P* < .001) than participants who did not perceive their weight as overweight (Table 3). Mean HDL cholesterol was 5.4 mg/dL lower among participants who perceived their weight as overweight than among those who did not (*P* = .02). Those who perceived their weight as overweight also had a 3.6 mm Hg higher diastolic blood pressure (*P* = .002) and an 18.4 cm higher mean waist circumference (*P* < .001) than those who did not. As in the unadjusted analyses, we found no significant associations between weight perception and HbA_1c_, fasting blood glucose, and systolic blood pressure.

**Table 3:** Cross-Sectional Linear Regression Results Between Weight Perception and Cardiometabolic Health Outcomes, NEXT PLUS Generation Health Study, US^a^

Biomarker	Did not perceive overweight	Perceived overweight	Difference	*P* value
**Adjusted for sex, race and ethnicity, socioeconomic status, and age**				
HbA_1c_, %	5.45 (0.07)	5.47 (0.07)	0.02 (.05)	.71
Fasting blood glucose, mg/dL	96.2 (2.0)	96.6 (1.9)	0.4 (1.4)	.77
High-sensitivity C-reactive protein, mg/L	0.96 (0.31)	2.10 (0.32)	1.14 (0.30)	<.001
Triglycerides, mg/dL	64.8 (6.8)	92.6 (8.8)	27.7 (10.9)	.01
Cholesterol, mg/dL	145.5 (4.8)	164.0 (6.1)	18.5 (6.5)	.005
High-density lipoprotein cholesterol, mg/dL	52.6 (1.6)	47.1 (2.0)	−5.4 (2.3)	.02
Low-density lipoprotein cholesterol, mg/dL	80.0 (4.2)	93.4 (4.3)	13.4 (3.4)	<.001
Systolic blood pressure, mm Hg	111.8 (1.3)	111.8 (1.4)	0 (1.3)	.98
Diastolic blood pressure, mm Hg	65.3 (1.3)	68.9 (1.3)	3.6 (1.2)	.002
Waist circumference, cm	76.3 (1.5)	94.7 (1.7)	18.4 (2.0)	<.001
**Additionally adjusted for body mass index**				
HbA_1c_, %	5.50 (0.07)	5.44 (0.07)	−0.07 (0.07)	.32
Fasting blood glucose, mg/dL	96.4 (2.1)	96.2 (1.8)	−0.25 (1.84)	.89
High-sensitivity C-reactive protein, mg/L	1.83 (0.36)	1.46 (0.35)	−0.37 (0.36)	.32
Triglycerides, mg/dL	79.1 (8.0)	84.2 (7.4)	5.0 (11.0)	.65
Cholesterol, mg/dL	150.0 (5.3)	162.3 (5.6)	12.3 (6.1)	.045
High-density lipoprotein cholesterol, mg/dL	49.2 (2.0)	49.5 (2.0)	0.3 (3.2)	.92
Low-density lipoprotein cholesterol, mg/dL	83.6 (4.4)	92.0 (4.2)	8.3 (4.0)	.04
Systolic blood pressure, mm Hg	111.9 (1.4)	111.9 (1.3)	0 (1.5)	.99
Diastolic blood pressure, mm Hg	66.5 (1.3)	68.1 (1.2)	1.5 (1.3)	.22
Waist circumference, cm	86.9 (0.8)	87.9 (0.7)	1.1 (1.0)	.30

a Values are adjusted mean (SE) unless otherwise indicated. Mean differences compare participants who perceived their weight as overweight with those who did not. All calculations used inverse probability weighting for wave 4. *P* values are from linear regression models.

In analyses additionally adjusted for BMI, adjusted mean total cholesterol was higher among participants who perceived their weight as overweight than among those who did not (162.3 mg/dL vs 150.0 mg/dL; mean difference, 12.3 mg/dL; *P* = .045). Adjusted mean LDL cholesterol was also higher among participants who perceived their weight as overweight compared with those who did not (92.0 mg/dL vs 83.6 mg/dL; mean difference, 8.3 mg/dL; *P* = .04). We found no significant associations between weight perception and HbA_1c_, fasting blood glucose, hs-CRP, triglycerides, HDL, systolic blood pressure, diastolic blood pressure, and waist circumference with additional adjustment for BMI.

### Longitudinal associations between baseline weight perception and cardiometabolic health 3 years later

In longitudinal models adjusted for sex, race and ethnicity, SES, age, and baseline cardiometabolic values, but not BMI, participants who perceived their weight as overweight had higher adjusted mean HbA_1c_ than those who did not (5.17% vs 5.08%; *P* = .05) (Table 4). Participants who perceived their weight as overweight also had lower adjusted mean LDL cholesterol than those who did not (89.4 mg/dL vs 98.9 mg/dL; *P* = .01). No other longitudinal associations were statistically significant. After additional adjustment for BMI, no longitudinal associations remained statistically significant.

**Table 4:** Longitudinal Linear Regression Results for Wave 4 Weight Perception and Wave 7 Cardiometabolic Outcomes, NEXT PLUS Generation Health Study, US^a^

Biomarker	Did not perceive overweight	Perceived overweight	Difference	*P* value
**Adjusted age, race and ethnicity, sex, socioeconomic status, and baseline cardiometabolic values at wave 4**				
HbA_1c_, %	5.08 (0.04)	5.17 (0.03)	0.08 (0.04)	.05
Fasting blood glucose, mg/dL	98.7 (1.6)	99.4 (1.6)	0.7 (2.1)	.75
High-sensitivity C-reactive protein, mg/L	2.13 (0.77)	1.78 (0.98)	−0.35 (1.40)	.80
Triglycerides, mg/dL	89.4 (6.4)	102.0 (9.9)	12.5 (10.7)	.25
Cholesterol, mg/dL	172.4 (4.7)	167.6 (4.4)	−4.7 (5.0)	.34
High-density lipoprotein cholesterol, mg/dL	53.8 (1.7)	53.1 (1.8)	−0.7 (2.0)	.70
Low-density lipoprotein cholesterol, mg/dL	98.9 (3.1)	89.4 (3.3)	-9.4 (3.6)	.01
**Additionally adjusted for body mass index**				
HbA_1c_, %	5.15 (0.04)	5.13 (0.04)	−0.02 (0.05)	.76
Fasting blood glucose, mg/dL	102.3 (1.8)	97.7 (1.6)	−4.5 (2.4)	.06
High-sensitivity C-reactive protein, mg/L	2.36 (0.66)	1.61 (0.89)	−0.75 (1.13)	.51
Triglycerides, mg/dL	95.4 (6.7)	100.6 (9.6)	5.2 (9.8)	.60
Cholesterol, mg/dL	169.5 (5.4)	168.8 (4.7)	−0.7 (6.5)	.92
High-density lipoprotein cholesterol, mg/dL	51.3 (1.8)	54.0 (1.8)	2.6 (2.1)	.21
Low-density lipoprotein cholesterol, mg/dL	97.0 (3.8)	89.9 (3.7)	−7.1 (5.2)	.18

a Values are adjusted mean (SE) unless otherwise indicated. All calculations used inverse probability weighting, using the longitudinal weight for waves 4–7.

## Discussion

We examined cross-sectional and longitudinal associations between weight perception and markers of cardiometabolic health in a nationally representative cohort of adolescents followed into emerging adulthood. In models adjusted for sociodemographic characteristics but not BMI, perceiving one’s weight as overweight was associated cross-sectionally with several less favorable cardiometabolic markers. After additional adjustment for BMI, perceiving one’s weight as overweight was still cross-sectionally associated with total cholesterol and LDL cholesterol, although no longitudinal associations remained statistically significant. These findings provide evidence that perceiving one’s weight as overweight is associated with worse cardiometabolic health, and further research is needed to understand the implications of these findings on clinical and public health recommendations aiming to notify individuals of their weight status.

In cross-sectional adjusted analyses, we found associations between perceiving one’s weight as overweight and elevated hs-CRP and diastolic blood pressure, and less favorable lipid profiles. Mean total cholesterol among participants who perceived their weight as overweight was higher than among those who did not and approached the upper normal limit of 170 mg/dL for this age group (29). Associations with total cholesterol and LDL cholesterol remained statistically significant after BMI adjustment; associations with hs-CRP, triglycerides, HDL cholesterol, diastolic blood pressure, and waist circumference did not. The elevations associated with perceiving one’s weight as overweight are concerning given that elevated cholesterol and LDL cholesterol levels are strongly associated with cardiovascular disease and strokes (29,30). Longitudinally, HbA_1c_ was slightly higher and LDL was lower among participants who perceived their weight as overweight in models not adjusted for BMI; neither association remained statistically significant after BMI adjustment. This may be because BMI partially confounds the longitudinal association between weight perception and cardiometabolic health. However, it is also important to consider the potential role of weight stigma. People with higher BMIs are not only more likely to perceive their weight as overweight, they are also subject to disproportionate weight stigma. Prior research suggests that weight stigma is associated with both psychological and physiological stress responses (4,31,32). A longitudinal study found that weight discrimination predicted increased allostatic load 10 years later, above and beyond the contributions of lifestyle and BMI (15). Thus, those with higher BMI who perceive their weight as overweight may demonstrate poorer cardiometabolic health because of both behavioral and biological manifestations of stress (31,32). Therefore, one hypothesis for our findings that warrants future study is that associations between weight perception and worse lipid levels may be at least partially attributable to an inflammatory response characteristic of chronic stress more than merely to BMI or health behaviors alone (33,34). Further research that examines the role of weight stigma in the association between cardiometabolic health and weight perception, above and beyond BMI and health behaviors, is warranted. In addition, analyses with larger samples should stratify results by BMI to see how results differ on the basis of actual BMI status, which would also affect their experiences of weight stigma or body dissatisfaction. Because many cross-sectional associations were attenuated after BMI adjustment, and only total cholesterol and LDL cholesterol remained associated with weight perception, our results do not show that perceiving one’s weight as overweight is associated with better cardiometabolic health. Public health and clinical policies are not the only potential drivers of weight perception (35). Future work should identify the factors that most strongly shape weight perception.

It is also possible we did not see more robust longitudinal associations between weight perception and cardiometabolic health because we adjusted for baseline cardiometabolic measurements taken at wave 4. If weight perception affects cardiometabolic health, effects may have already manifested by wave 4; alternatively, given the young age of the sample, cardiometabolic markers may not have varied enough over the 3-year follow-up to detect change. A 3-year follow-up period may also have been too short to detect longer-term associations between weight perception and cardiometabolic health. Unger et al found longitudinal associations between perceiving one’s weight as overweight in adolescence and higher blood pressure 12 years later among adolescents with higher body weights (13). Nonetheless, our cross-sectional findings especially raise concerns about prevailing practices and policies that encourage BMI reporting. Although these efforts are intended to improve health outcomes, our results provide evidence that perceiving oneself as overweight is actually associated with worse cardiometabolic profiles; further prior literature suggests that perceiving one’s weight as overweight can be harmful for other behavioral and psychological outcomes. Thus, we urge caution toward blanket public health initiatives that aim to inform adolescents of their weight status, because the literature suggests that such practices may have negative mental and physical effects for some adolescents during a critical time in development.

### Strengths and limitations

A notable strength of our study is the use of objective anthropometric and cardiometabolic measures. In addition, we used longitudinal data from a nationally representative cohort of adolescents followed into emerging adulthood, allowing us to examine temporal associations for outcomes measured at wave 7. However, our sample size was limited because biomarker data were available only for a subsample of participants, reducing power for subgroup analyses and increasing the need to interpret marginal *P* values cautiously. Given the sample size, we could not stratify results based on sociodemographic factors such as race and ethnicity, sex, or BMI. We examined multiple cardiometabolic outcomes, and *P* values were not adjusted for multiple comparisons; therefore, results should be interpreted cautiously and replicated in larger samples. Because experiences and meanings of weight perception may differ by these factors, associations between weight perception and cardiometabolic outcomes may also differ across groups. For example, larger bodies among women are seen as more negative than larger bodies among men, and therefore, weight perception may more negatively affect physiological health for women compared with men (36). Moreover, people with higher BMI may experience more weight stigma, which could adversely affect cardiovascular health through behavioral or stress-related pathways. Therefore, future research should investigate whether associations between weight perception and cardiometabolic risk differ across these sociodemographic factors. In addition, because we were not able to differentiate between weight perception and weight stigma or body satisfaction in the present study, future research should use validated measures of weight stigma experiences, weight-bias internalization, and body dissatisfaction in addition to weight perception to explore the independent contributions of each of these factors to cardiometabolic health. Finally, the blood pressure and waist circumference data were not available at wave 7, which precluded longitudinal analyses for those outcomes.

### Conclusions

In this nationally representative cohort of adolescents followed into emerging adulthood, perceiving one’s weight as overweight was associated with less favorable cardiometabolic markers in cross-sectional models but not longitudinally. Cross-sectional associations of weight perception with total cholesterol and LDL cholesterol remained statistically significant after BMI adjustment. Overall, findings do not support the assumption that perceiving one’s weight as overweight is associated with better cardiometabolic health. Health professionals should consider the possible implications of policies aimed at informing young people of their BMI status, because such policies may have counterproductive or even harmful effects for some. Future studies should use larger samples, repeated cardiometabolic measurements, and validated measures of weight stigma, weight-bias internalization, body satisfaction, and health behaviors to clarify mechanisms linking weight perception and cardiometabolic health.
